# Tumor necrosis factor-alpha inhibitors may increase the need for fine-needle aspiration cytology in axial spondyloarthritis patients

**DOI:** 10.1590/1806-9282.20240871

**Published:** 2024-12-02

**Authors:** Zeynel Abidin Sayiner, Hayriye Sultan Munis, İpek Köroğlu, Elif Melis Baloğlu Akyol, Orhan Zengin, Ersin Akarsu

**Affiliations:** 1 Gaziantep University, School of Medicine, Department of Endocrinology and Metabolism – Gaziantep, Turkey.; 2 Gaziantep University, School of Medicine, Department of Internal Medicine – Gaziantep, Turkey.; 3 Gaziantep University, School of Medicine, Department of Rheumatology – Gaziantep, Turkey.

**Keywords:** Anti-inflammatory agents, Fine-needle aspirations, Thyroid nodules, Tumor necrosis factor inhibitors

## Abstract

**OBJECTIVE:**

Tumor necrosis factor alpha inhibitors are frequently used in the treatment of axial spondyloarthritis. While tumor necrosis factor alpha is associated with some malignancies, studies on its effects on thyroid functions and thyroid nodules are limited. This study aimed to assess the effect of tumor necrosis factor alpha inhibitors treatments on the evaluation of thyroid nodules and thyroid function tests in axial spondyloarthritis patients.

**METHODS:**

A total of 106 patients with axial spondyloarthritis, 48 receiving nonsteroidal antiinflammatory drugs and 58 receiving tumor necrosis factor alpha inhibitors, were included in the study. All cases were screened by ultrasound for the presence of nodules, European Thyroid Association – Thyroid Imaging Reporting and Data System classification category, thyroid volume, and thyroid function tests.

**RESULTS:**

The prevalence of patients with multiple nodules in the tumor necrosis factor alpha inhibitors treatment group was significantly higher compared to the nonsteroidal antiinflammatory drug group (68.2 vs. 38.1%, p=0.033). Furthermore, the prevalence of nodules measuring ≥1 cm in the tumor necrosis factor alpha inhibitors treatment group was higher compared to the only nonsteroidal antiinflammatory drug treatment group (56 vs. 19.05%, p=0.011). In the tumor necrosis factor alpha inhibitors treatment group, the proportion of patients with nodules requiring fine-needle aspiration cytology was higher compared to the nonsteroidal antiinflammatory drug group (34.5 vs. 16.7%, p=0.038). No significant difference was found between thyroid-stimulating hormone and free thyroxine values before and during treatment in both groups (p>0.005).

**CONCLUSION:**

The number of nodules requiring fine-needle aspiration cytology according to the European Thyroid Association – Thyroid Imaging Reporting and Data System classification seems higher in nonsteroidal antiinflammatory drugs users than Tumor necrosis factor-alpha inhibitor users. Therefore, it may be rational to examine the thyroid gland while administering Tumor necrosis factor-alpha inhibitor treatment in axial spondyloarthritis patients.

## INTRODUCTION

Spondyloarthropathies (SpA) are chronic inflammatory diseases primarily affecting the spine and sacroiliac joints. Ankylosing spondylitis is the most common form of spondyloarthropathy^1^. Axial spondyloarthropathy (Ax-SpA) is classified into patients with and without radiographic axial findings. Nonsteroidal antiinflammatory drugs (NSAIDs) are used as the primary treatment for Ax-SpA. Anti-tumor necrosis factor (TNF) drugs have emerged as the primary treatment option for Ax-SpA patients who do not improve with NSAIDs^2^. Tumor necrosis factor-α inhibitors (TNFαi) such as adalimumab, certolizumab pegol, etanercept, golimumab, and infliximab have had a major impact on the treatment of ankylosing spondylitis (AS) over the last two decades^3^. Anti-TNF therapy has been associated with an increased incidence of infection and malignancy and the worsening of existing heart failure^4^. Some studies suggest that the incidence of cancer, other than lymphoma, has not changed significantly, while other studies show an increased risk of solid tumors and skin cancer^1^. A recent study reported that the likelihood of developing hematological malignancies in patients with inflammatory arthritis who were treated with TNFαi was higher^5^. Before starting TNF-α inhibitor therapy, it is recommended to undergo age-appropriate cancer screening tests, including a physical examination, chest X-ray, fecal blood test, breast examination, vaginal pap smear, lung and heart auscultation, as well as a lymph node examination^6^. Screening for thyroid cancer is not routinely performed in patients scheduled to start TNFαi therapy. Human thyrocytes have a unique receptor for TNF-alpha. TNF-alpha exerts both paracrine and endocrine effects on the inflammatory and immune systems and can function independently. Together with other cytokines, TNF-alpha is probably involved in the localized control of thyroid function^7^. Whether this exists physiologically or only in pathological conditions in the thyroid has not yet been established. Limited studies evaluate thyroid function tests in Ax-SpA patients on TNF-alpha inhibitors, and only a few studies analyze the effect of TNF-alpha inhibitor use on thyroid nodules and its association with thyroid cancer. Understanding these effects is crucial for developing appropriate monitoring and management strategies for the patients. This study aimed to evaluate the effect of TNF-alpha inhibitors on the evaluation of thyroid nodules and thyroid function tests in patients with axial spondyloarthritis.

## METHODS

The study had a retrospective, cross-sectional, and non-interventional design. Axial spondyloarthropathy was diagnosed using the Assessment of SpondyloArthritis International Society classification criteria^8^. A total of 106 patients were included in the study. Patients diagnosed with axial SpA between 2005 and 2023, whose thyroid function tests were available at diagnosis and who were currently followed up in our center, were included. The study included patients diagnosed with Ax-SpA who had been prescribed NSAIDs or a single anti-TNF therapy for a minimum of 1 year. Participants were between the ages of 18 and 65 years, with no documented history of cancer or thyroid disease, and who had not undergone FNAC for thyroid nodules in the past. They were checked for thyroid nodules in the past from health system records and had undergone thyroid ultrasound within the last 10 years with no thyroid nodules. The study excluded patients with a history of irregular follow-up, diagnosed thyroid disease previously, anti-thyroid peroxidase antibody positivity, anti-thyroglobulin antibody positivity, radiation exposure to the neck region, or those taking medications that could impact thyroid function, such as oral contraceptives, amiodarone, somatostatin analogs, lithium, iodine, multivitamin tablets, corticosteroids, and sulfasalazine within the last 10 years. Patients were evaluated in two groups: 48 axial SpA patients receiving NSAIDs since diagnosis and 58 patients who initially took TNF-alpha inhibitors (etanercept, infliximab, adalimumab, golimumab, and certolizumab). Various factors, including patient demographics, disease duration, current medications and their duration, HLA B27 results, and anti-thyroid peroxidase levels, were assessed. Thyroid function tests, C-reactive protein, sedimentation rate, and white blood cell counts were also examined. Thyroid ultrasounds were performed on all patients using a 7–12 MHz linear probe (GE-Logic P9) by two distinct expert endocrinology specialists to ensure unbiased measurements. Thyroid parenchyma, thyroid volume, and thyroid nodules were extensively assessed. The length × depth × width × π/6 formula was used to calculate thyroid volume^9^. The study obtained approval from the university’s Human Ethical Board Committee (number: 2023/119). All participants signed informed consent forms.

### Statistical analysis

Data were analyzed using descriptive statistics. Numerical variables were summarized using mean, standard deviation, median, minimum, and maximum values. Categorical variables were analyzed using frequency and percentage. The Shapiro-Wilk test was used to analyze the compatibility of variables with normal distribution. Comparative statistical analysis was conducted using either the independent samples t-test or the Mann-Whitney U test. The Wilcoxon test was used to compare TSH and FT4 values before and after treatment within each group. The Spearman correlation analysis was employed to ascertain the correlation between disease duration and thyroid volume. Chi-square analysis tested differences between categorical variables. Analyses were conducted using the SPSS 22.0 software. A significance level of p<0.05 was selected.

The TNFαi group had 58 patients, and the NSAID group had 48 patients. The anti-TNF group had 37 (63.79%) men, while the NSAID group had 30 (62.5%) women. The mean age of the anti-TNF group was 51.1±11.01 years, and the mean disease duration was 9.74±4.11 years. The mean age of the NSAID group was 43.17±14.34 years, and the mean disease duration was 4.98±3.73 years. The distribution of TNF-alpha inhibitor use was as follows: adalimumab 12 patients, etanercept 12 patients, golimumab 12 patients, infliximab 12 patients, and certolizumab 10 patients. No difference was found between the TNFαi group and the NSAID group in terms of white cell count, C-reactive protein, erythrocyte sedimentation rate, TSH, and free T4 values. The biochemical values at the first diagnosis of SpA are shown in Table 1. Baseline and current thyroid function tests of both groups were compared, with no significant difference found in TSH and free T4 values after 1 year of initial treatment (Table 1).

**Table 1:** Biochemical values of the groups before the first diagnosis of axial spondiloarthropathy.

Variables	TNF-α inh Group n=58		NSAID Group n=48		p
	Mean±SD	Median (Q1–Q3)	Mean±SD	Median (Q1–Q3)	
White blood count (×10^9^/L)	8.4±2.7	8.0 (6.5–9.6)	7.3±1.7	7.3 (5.9–8.5)	0.051^a^ *
C-reactive protein (mg/dL)	6.45±9.02	4.0 (1–8)	6.2±5.9	4 (2–9)	0.493^a^
Erythrocyte sedimentation rate (mm/h)	23.3±17.6	20.5 (11–31)	18.4±9.6	17 (12.5–25)	0.298^a^
TSH level before diagnosis (mU/mL)	1.72±1.44	1.2 (1.0–2.1)	1.7±0.9	1.7 (1.3–2.3)	0.934^b^
fT4 level before diagnosis (ng/dL)	0.88±0.26	0.8 (0.8–0.7)	0.7±0.1	0.8 (0.8–0.8)	0.392^b^

fT4: free thyroxine; SD: standard deviation; TSH: thyroid-stimulating hormone; TNF-α inh: tumor necrosis factor alpha inhibitors.

*p<0.05.

^a^Mann-Whitney U test;

^b^Student’s t-test.

While no difference was found between the groups in the presence of nodules, nodule echogenicity, and thyroid gland heterogeneity, the percentage of patients with multiple nodules in the anti-TNF group was higher than in the NSAID group (69.2 vs. 38.1%, p=0.033) (Table 2). Additionally, the number of nodules with the longest diameter ≥1 cm was higher in the TNFαi group than in the NSAID group (57.8 vs. 19%, p=0.011). According to FNAC results, no malignancy or suspected malignancy was detected in the Bethesda classification in both groups. Although no difference was found between the groups in terms of disease duration, presence of thyroid nodules, and thyroid gland volumes, there was a weak positive correlation between disease duration and thyroid gland volume (p=0.031, r=0.209). Thyroid volume increased with disease duration. The proportion of patients with nodules requiring FNAC according to the European Thyroid Association-Thyroid Imaging Reporting and Data System was higher in the anti-TNF group than in the NSAID group (50 vs. 19%, p=0.028) (Table 3).

**Table 2:** Comparison of thyroid ultrasound characteristics of treatment groups.

Variables		TNF-α inh n (%)	NSAID n (%)	p
Thyroid gland	Homogeneous	20 (35.1)	24 (50)	0.107
	Heterogeneous	28 (65.5)	24 (50)	
Nodule presence	Present	26 (44.8)	21 (43.75)	0.911
	Absence	32 (55.1)	27 (56.25)	
Nodule count	Solitary	8 (30.8)	13 (61.9)	0.033*
	Multiple	18 (69.2)	8 (38.1)	
Echogenicity of the nodule	Hypoechoic	8 (30.8)	7 (33.3)	0.691
	Isoechoic	1 (3.8)	2 (9.5)	
	Hyperechoic	0 (0)	0 (0)	
	Mixed echoic	17 (65.4)	12 (57.1)	0.766
Longest nodule diameter	<1 cm	11 (42.3)	17 (80.9)	0.011*
	≥1 cm	15 (57.8)	4 (19.0)	
EU-TIRADS classification of nodules	EU-TIRADS 1	5 (10.4)	17 (35.4)	0.018*
	EU-TIRADS 2	14 (29.1)	16 (33.3)	
	EU-TIRADS 3	17 (35.4)	6 (12.5)	0.021*
	EU-TIRADS 4	11 (22.9)	9 (18.5)	
	EU-TIRADS 5	1 (2.0)	0 (0)	

TNF-α inh: tumor necrosis factor alpha inhibitors; EU-TIRADS: European Thyroid Association – Thyroid Imaging Reporting and Data System; NSAID: nonsteroidal antiinflammatory drugs.

*p<0.05.

**Table 3:** Fine-needle aspiration cytology requirement status of groups according to European Thyroid Association – Thyroid Imaging Reporting and Data System recommendations.

		TNF-α inh n (%)		NSAID n (%)		p
		n	n%	n	n%	
FNAC requirement according to EU-TIRADS recommendations	Yes	13	50	4	19	0.028*
	No	13	50	17	81	

*p<0.05 is significant; Chi-square test; FNAC: fine-needle aspiration biopsy; EU-TIRADS: European Thyroid Association – Thyroid Imaging Reporting and Data System; TNF-α inh: tumor necrosis factor alpha inhibitors.

## DISCUSSION

Understanding the impact of TNFαi treatment on thyroid function and nodule formation is crucial in providing comprehensive care for patients receiving this therapy, particularly those with autoimmune conditions like axial spondyloarthropathies. Various studies have highlighted potential associations between TNFαi therapy and thyroid-related complications, such as alterations in thyroid function and an increased risk of nodules. A study by Paschou examined the impact of TNF-alpha inhibitors on thyroid function, revealing a decrease in free thyroxine (FT4) levels among patients, though no alterations were observed in TSH, triiodothyronine (T3), or thyroid autoantibodies. There were also suggestions that TNF-alpha has an inhibitory impact on type 2 deiodinase. TNFαi therapy may reduce the D2 deiodinase-suppressing effect of TNF-α, leading to more T4 to T3 conversion in the periphery, resulting in a decrease in free T4^10^. A meta-analysis evaluating the effect of anti-TNF agents on thyroid function in individuals with rheumatological diseases analyzed 1,224 patients from 16 randomized controlled trials. The results showed that FT4 levels were significantly reduced in patients receiving TNFαi therapy, though no significant changes were found in TSH and T3 levels^11^. It should be noted that most patients in this meta-analysis had rheumatoid arthritis, with a small number diagnosed with Axial SpA. NSAIDs can increase free T3 and T4 levels by affecting the binding of thyroid hormones, leading to higher levels in thyroid test results. However, each individual’s response to NSAIDs may vary, affecting thyroid test results differently^12^. The number of studies evaluating thyroid function tests in patients with axial spondyloarthropathy and taking appears to be significantly small. In this study, thyroid function tests of both groups were within normal limits, and there was no significant difference between the two groups.

The prevalence of thyroid nodules varies based on factors such as age, sex, radiation exposure, and iodine deficiency^13^. In one study, the effects of TNFαi on thyroid nodules were analyzed. Thyroid nodules were found in 38.5% of patients receiving TNFαi and 36.7% of patients not receiving. There was no statistically significant difference between these two groups. However, the risk of thyroid cancer was found to be higher in patients receiving TNFαi compared to those not receiving TNF-alpha inhibitors, according to FNAC results^14^. In our study, the presence of thyroid nodules was slightly higher in TNFαi users (44.8 vs. 43.75%; p>0.05). The prevalence of thyroid nodules in adults in the geographical area of this study was found to be 23.5% by ultrasonography, indicating that thyroid nodules may be more common in Ax-SpA patients. Additionally, the number of nodules requiring FNAC and the presence of multiple nodules were more common in the group receiving TNFαi. However, there was no difference in the detection of malignancy according to BETHESDA categories in patients who underwent FNAC. However, it has not been determined whether this condition during treatment is due to the drug, the natural course of the disease, or coincidental. While more nodules were observed in TNFαi treatment recipients compared to NSAID recipients, the fact that there was no difference in terms of malignancy development may be due to the limitations of our study. Studies with longer duration and more unified groups may reveal clearer results. Well-designed case-control or cohort studies are needed to evaluate the risk of cancer in patients receiving TNFαi therapy.

The limitations of the study include its single-center design, lack of control for patients’ iodine intake status, lack of a control group of healthy individuals, and a relatively small sample size. The time interval between the start of TNFαi treatment and thyroid ultrasound was not clearly known for each patient.

## CONCLUSION

Axial spondyloarthropathy patients using TNF-alpha inhibitors may have thyroid nodules more frequently than NSAID users. Patients using TNF-alpha inhibitors have a higher number of thyroid nodules and a higher frequency of nodules requiring FNAC according to EU-TIRADS criteria. TNF-alpha inhibitor use does not affect thyroid function tests. It should be considered that thyroid nodule evaluation may be necessary before starting TNF-alpha inhibitors and during treatment. However, well-designed case-control or cohort studies are needed to evaluate the risk of cancer in patients receiving TNFαi therapy.
